# Towards Open Information Management in Health Care

**DOI:** 10.2174/1874431100802010042

**Published:** 2008-03-24

**Authors:** J Yli-Hietanen, S Niiranen

**Affiliations:** Department of Signal Processing, Tampere University of Technology, Finland

## Abstract

The utilization of information technology as tool in health care is increasing. The main benefits stem from the fact that information in electronic form can be transferred to different locations rapidly and from the possibility to automate certain information management tasks. The current technological approach for this automation relies on structured, formally coded representation of information. We discuss the limitations of the current technological approach and present a viewpoint, grounded on previous research and the authors’ own experiences, on how to progress. We present that a bottleneck in the automation of the management of constantly evolving clinical information is caused by the fact that the current technological approach requires the formal coding of information to be static in nature. This inherently hinders the expandability of the information case space to be managed. We present a new paradigm entitled open information management targeting unlimited case spaces. We also present a conceptual example from clinical medicine demonstrating open information management principles and mechanisms.

## INTRODUCTION

Looking back at the history of health informatics, the use of information technology in health care began with the use of computers to automate large, repetitive and well-defined tasks related to billing, bookkeeping and inventory management and similar simple workflows. It is widely accepted that the adoption of automated computation to replace actuaries brought time and cost savings. As these basic logistical tasks were gradually automated with observed benefits, reflecting the similar developments in other areas of the economy, the obvious next step within the emerging health informatics community was to automate clinical data management and to provide support for clinical workflows. However, Dorenfest summarizes that investments in computerized patient records have not been accomplishing their objectives [1]. Also, adoption and diffusion rates for both inpatient and outpatient electronic health records are reported to be low [2,3]. Health informatics systems successes and failures have been further analyzed [4,5]. In spite of demonstrated benefits, there are more severe problems than reports on successes suggest [5] and health informatics systems failures have been characterized as a significant problem [4]. In relation to this, it has been reported [6, 7], that in some cases the adoption of computerized systems may even produce errors instead of reducing their likelihood.

There have been numerous attempts at molding conventional information technology to fit better to clinical workflows through various modeling based approaches:

1. Standardization efforts implementing unified concept hierarchies and ontologies for common semantics and common information exchange standards to provide system level interoperability [8-10]. The goal has been to provide seamless support for clinical workflows by providing tools for integrating smaller systems supporting parts of the process in question.
2. Development of rapid application development methodologies for clinical processes [11, 12]. The goal has been to provide tools for rapidly implementing and augmenting systems supporting complex and evolving workflows.

These attempts at molding conventional information technology to support clinical workflows have had their ultimate goal in the conventionally understood electronic health record. However, there is no general agreement what an electronic health record is and not even one single mutually agreed name for it. This reflects the ambiguity of the concept. One is tempted to hypothesize that the conventionally understood electronic health record as a static, comprehensive and formal definition is not an optimal approach as this type of modeling may prove to be prohibitively complex in reflecting the reality of clinical work. This problem area is further discussed in [13]. For example, to be able to define an ideal electronic health record, the entire health care system would have to be modeled including all the underlying behaviors hidden in medicine and human activities. Due to this, the utilization of technologies such as conventionally implemented computer-interpretable clinical guidelines [14] is laborious within clinical workflows as a relatively accurate prediction of usage scenarios is typically required. In reality, the information case space of a typical clinical workflow is constantly evolving and expanding.

Recent discussion in health informatics has emphasized support of dynamic clinical communication in contrast to emphasis on information representation and storage as well as on information system design culminating in the conventional concept of an electronic health record [15]. Up to 90% of clinical information transactions in some environments do not involve stored electronic data, but are rather information exchanges, often made face-to-face, between clinicians [16]. Furthermore, it is argued in [15] that: “Communication systems are a crucial component of the information infrastructure of any health care organization, not just as pipes through which information flows, but as the systems where humans share, discuss and eventually decide upon clinical actions.” This communication and interaction oriented paradigm of clinical information management is further expanded in [17, 18].

As we have catalogued the major complexities faced in the development of a comprehensive electronic health record, the question might arise why information technology is needed to support dynamic clinical communication? After all, face-to-face communication between clinicians works naturally, easy-to-use telephones are available for location independent communication and so on. According to Toussaint and Coiera the increasing complexity of health care processes creates a demand for communication support tools [15]. Extending from [15], these complexities emerge from the increasing number of organizations and personnel who work together to provide care for an individual patient and from an increasing need for cost efficiency and advances in medical science. This creates an avalanche of changes in policy and practice which need to be widely disseminated. On the other hand, modern management theory has raised the potential of personnel empowerment in work method improvement, also in health care [19]. Ultimately, this implies that personnel at every level of an organization should be able to contribute, for example, to the development of tools used, including those related to communication.

Conventional natural conversations and basic electronic communication tools are not up to the challenge. As the intensity of communication increases, use of synchronous communication, such as face-to-face or telephone conversations, becomes prohibitively time consuming and disorienting. Conventional tools for asynchronous communication, such as e-mail, do provide means for time and location independence but they do not provide the sender with an instant possibility to sense that the recipient understands the meaning of the message. Thus, the time needed to coordinate an activity *via *e-mail typically takes more time than using a phone since the context of the message has to be more verbose. Also, information contained in or emerging from natural conversations is often laborious to discover by third parties as it remains only as the personal knowledge of the original communicators. Typically, such knowledge can only be retrieved by asking the original communicators. However, it can be argued that third parties may not even be aware that a relevant piece of clinical information is available for them to ask.

Thus, support of dynamic clinical communication should be one starting point for developing tools capable of fitting with the complexities eminent in clinical work. Novel technologies, departing from simple conventional communication tools, are needed to make clinical communication truly dynamic and congruent with clinical work. Since the notion of dynamic communication as key tool in clinical informatics was raised, a number of system implementations emphasizing support of clinical communication have been presented, e.g. in [20].

As illustrated by the surveyed previous research, there exists a wide range of challenges in health informatics. The identified primary challenges are evolving and expanding information case spaces of clinical workflows and the related need for tools supporting communication in changing and varied clinical settings. In this paper, we present a new paradigm entitled open information management targeting unlimited case spaces, which provides native support for dynamic clinical communication.

## OPEN INFORMATION MANAGEMENT

Considering the history of patient records, a trend towards the use of structure to organize information is easy to observe. The first medical records were basically hand-written notes on paper. As printing technology developed, increasingly structured forms were taken into use to handle information management tasks in various medical situations and specialties. Of course, concurrent to this development, and a driving force behind it, was the increasing specialization and complexity of medicine. As the shift towards an electronic patient record began, it can be argued that the electronic form actually increased the use of rigid structures to a level where it began to hinder the natural paths of clinical communication. For example, structural paper forms, which help personnel to remember to write down the required information, still allowed the inclusion of ad-hoc and free-form comments on the document (e.g., on the backside of the paper). Of course, due to this, there are free-text comment fields in electronic documents to complete a certain piece of structured information. However, the allowance of free-text completions and annotations hinders the automation of information management, which was the original goal of the use of electronic systems. This is due to the fact that current information management technology relies on a paradigm where real automation is only possible with formal, rigid processing rules relying on the use of pre-designed structures for information containment.

Current information technology does help to replace the conventional archivist and document courier with a remarkable increase in the efficiency of copying and distributing documents. Also, reporting and other aggregative forms of information processing have become more efficient through the fast computation of highly structured data available from electronic documents. Furthermore, it has provided increased automation of information management when the clinical application domain is relatively static and can thus be captured in designs fixed before use. However, there are many clinical application areas that appear to be difficult for automation with the design-before-use paradigm due to a constantly changing clinical operational environment. The limitations of this paradigm have received attention in the context of medical expert systems. Ripple-down Rules knowledge acquisition technology was initially developed to deal with the maintenance problems of medical expert systems [21]. It is an approach to building knowledge-based system incrementally, while the system is in routine use by utilizing the contextual nature of expert knowledge [22, 23].

Next, we present a set of basic paradigms representing our rethinking of clinical information management. The paradigms constitute the foundation for open information management. By ‘open’ we mean that the case space of the information to be managed is principally unlimited. This is in contrast to conventional information technology where the required formalization of the information inherently hinders the expandability of the information case space. Conventionally, this limitation has not been a disadvantage since information technology has been applied to situations where the use scenarios have been assumed fully known before-hand. On the other hand, the limited case space enabled the design and completion of information management systems before deployment into use. As the case space of information is often unlimited (i.e., open), systems managing such information cannot be completed before use. The fact that an open information management system has to evolve during use, changes the role of the system from just being a passive tool towards an active member in collaboration. The difference in the approaches could be exemplified in that while the former warehouses documents without understanding the content, the latter cumulates contextualized pieces of information. Naturally, the role of an information management system as a collaborating partner is significantly different from that of human partners. We are not proposing the re-invention of artificial intelligence [24] having the ultimate goal in fully replacing people in selected activities. With open information management we target the maximization of the level of assistance the technology is able to deliver.

## FOUNDATION

How do we automate a task? We create an automated action that results in the completion of the task. Thus, automation of an information management task is the creation of an automated action resulting in processed information corresponding to the goal of the task. The goal of the task might, for example, be to make a selected group of people aware of a change in certain situation relevant to their work. It is important to note that completion of the task is essential, not how it was achieved. Another key point is that the fulfillment of the goal is an unambiguous sign of the completion of the task.

At this stage it is sobering to consider the current way how information technology strives towards the automation of information management. The goal of the task is taken as the starting point for a system design process. This goal is then mapped into functionalities of system. After the functionalities of the system have been fixed, an implementation and testing process is carried out resulting in a system reflecting the mapped functionalities. It is important to note that the goal is not explicitly contained in the implementation of the system. Thus, when the goal changes a remapping from the goal to the functionalities has to be made. The fundamental observation here is that the mapping between the goal and the functionalities is typically complex, meaning that a relatively minor change in the goal can result in numerous changes in functionalities.

The current mainstream approach to enhance the response to goal evolution has been development acceleration or goal generalization. In the former approach, tools are developed to accelerate the transformation of the goal into system functionalities while the latter approach is to base a system design on a more general goal leaving the user with more degrees of freedom. While neither approach changes the basic fact that the goal is not explicitly contained in the implementation of the system, they do provide for enhanced systems engineering. However, our viewpoint is that goal-orientation should be native to the implementation instead of current functionality-orientation. Our opinion is that this shift should provide for enhanced adaptation to changes in the goal as goal dynamics are directly connected to and guiding the operation of the system. The obvious next question is how goal-orientation can be made native to the system implementation. We will begin the process by first briefly looking at human collaboration.

Considering collaboration among health care professionals, goal-orientation is native to the process. What means are leveraged in this goal-oriented process? First of all, coordination of collaboration is natively carried out in natural language communications. As linguists have pointed out [25], this coordination of collaboration relies on the existence of a common ground between collaborators. Knowledge accumulated from shared past experiences, or available through a similar background, is the basis of a common ground. Obviously, each joint instance of collaboration increases the shared common ground. This makes coordination of collaboration increasingly efficient. How is the accumulation of knowledge based on experiences possible? Each new experience has to be somehow connected to previous experiences in order for us to understand its meaning in relation to the goal of collaboration. Characteristic of these connections is that potentially everything relates to everything.

As we are working towards making goal-orientation native to an information management system, a key observation is that use of such a goal-oriented system resembles more collaboration towards a goal. This is in contrast to the current paradigm where users are restricted to before-use-designed functionalities whose evolution is constrained by the latency and resource use caused by the separate process required to change the functionalities.

We will next look at some fundamental requirements arising from goal-orientation. Naturally, there has to be a way to express the goal for the system and to continuously refine it. Taking a cue from how clinicians utilize an increasing common ground to efficiently coordinate their collaboration, we also need a way to reuse previously carried out operations as a similar kind basis for new operations. As we wanted the system to perform automated information management actions, there has to be a way to connect the actions, the goal and the information to be managed into an operational entity.

## OPERATION

As we have now discussed at an abstract level the foundational paradigms for open information management, we will move on to more operational aspects of a system performing goal-oriented information management tasks.

First of all, we begin by noting that information management automation is only possible when the information to be managed is contextualized. In a functionality-oriented information management system, information is contextualized by inserting it into a structure designed before use. As a point of observation, this way of contextualization has severe drawbacks when it comes to situations where the goal is changing. The key fact is that functionality based on rigid rules, providing automation, are rigidly dependent on corresponding structure. The result is that when functionality changes, information structures have to be transformed correspondingly. Thus, the problem of legacy information creates complexities in the evolution of functionality. To prevent this, contextualization in goal-oriented information management should not rely on rigid, pre-designed structure.

If we leave pre-designed structure behind, how do we enable information management automation? We propose that information is contextualized by augmenting it with explanations during use. In order to fully avoid pre-design, it cannot be assumed that explanations are complete before information management actions begin. The implication is that the accompanying information management automation system has to be able to handle incompletely contextualized information.

How can the explanations enable information management automation? We propose to leverage the recent research on semantic networks [26] and on the use of semi-structured natural language fragments as a knowledge representation mechanism [27] as a point of reference. Whereas semantic networks target general textual reasoning, we look at possibilities of semantic connectivity as an enabling mechanism. Specifically, we propose that the explanations describing information to be managed should be semantically connected to explanations describing available actions and to explanations describing the desired result of information management (i.e., the goal).

What is the nature of the semantic connections relating the explanations to each other? First of all, the basic element in all explanations is a concept expressed by a single word. As soon as a new explanation contains a word contained in another explanation, they are immediately semantically connected *via *that word. A key observation is that implicit connections emerge automatically without any separate effort or structure. This is in contrast to the functionality-oriented approach where only explicit mechanisms are used to connect pieces of information to functionalities.

What is the vocabulary for the words in the explanations? To avoid the need for pre-design, the vocabulary cannot be fixed before use but rather it should expand continuously as new words appear in explanations. As explanations describing, e.g., the goal are given by users and are related to the work and the physical working environment, vocabulary of a natural language is a well-grounded choice.

Going back to the question of how to enable information management automation, we propose that this is achieved by complementing the explanation of the goal until it is semantically connected to specific actions and to the information to be managed. On a more practical level, the explanation of the goal has to create an execution path defining how the information to be managed gets processed by specific actions. To be able to form an unambiguous execution path, the connections between concepts in a single explanation have to be explicit. We propose that such an explicit connection could be a direct link between two concepts.

Automation is achieved when the system is given new information to be managed and a goal matching to a previously given and subsequently fulfilled goal. In this specific case, the system can repeat the actions fulfilling the goal with the given new information. Also, even if there is no direct match between a previous goal and a given goal, semantic matching can be leveraged to partially reuse previously given explanations. This reuse can be seen as corresponding to the concept of common ground utilized in linguistics. Considering the reuse of explanations, it is obviously required that earlier explanations are accessible for later use. It should be thus noted that multiple explanations for the same thing will unavoidably exist. This does not present a problem but is rather an asset as the idea is to leverage previous explanations as a kind of salvage yard for the creation of new explanations.

One aspect of the proposed goal-oriented approach is that complementing the explanation of the goal can also lead into the introduction of new actions. The key observation here is that making a new action available does not require any changes in previously given explanations. This is contrast to functionality-oriented systems where the introduction of a new functionality typically creates multiple and interconnected changes in information containment structures and subsequently in other functionalities.

Ambiguities, incompleteness and inconsistencies have conventionally caused difficulties and complexities for functionality-oriented systems. This stems from the fact that information management automation in these systems relies on the use of rigid, pre-designed structures and rules that can not handle the unexpected. Goal-orientation does not rely on pre-design but rather on the complementation of explanations on an on-demand basis, during use. Thus, ambiguities, incompleteness and inconsistencies are not a threat to be carefully avoided but rather an unavoidable characteristic of the operation environment and are tackled by the user complementing the explanations.

## ANTICOAGULATION TREATMENT

Anticoagulation treatment is indicated by a number of conditions, the most common of which is chronic atrial fibrillation [28]. An orally taken blood thinning medication is followed up regularly with the measurement of the P-INR index which basically measures blood thickness. The typical chronic atrial fibrillation patient has a treatment range of 2 – 3 P-INR. The measured P-INR value and a patient’s care history are evaluated to arrive at new daily dosages for the orally taken anticoagulant and the next follow-up date. In the public Finnish care delivery system, routine anticoagulation treatment follow-up is organized by primary health care centers. The treatment is typically controlled by general practitioners, which involves the evaluation of P-INR results and setting new treatment guidelines. In the context of regional primary care practice development, new work and information management strategies involving point-of-care testing and devolution of routine duties to selected anticoagulation nurses have been studied [29]. One starting point in this study was the controlled devolution of guideline assignment duties to physicians. This was supported with the introduction of an electronic anticoagulation task management and notebook system whereby a single nurse could observe new follow-ups as they become available from a laboratory information system, forward deviating results for review by physicians and assign guidelines for routine follow-ups. Considering the topic of the current study, we will next consider the observed characteristics of anticoagulant treatment follow-up information management as a case for open information management. We hope to bring forward some of the possibilities offered by the open approach when compared to the typical information management tools available. The aforementioned earlier study on anticoagulation treatment management by the authors utilized a conventional IT support tool and this study leveraged data and experiences from it.

One characteristic of anticoagulation treatment follow-up, in a setting where care information management is primarily carried out by a single anticoagulation nurse, is the principally boundless number of ways in which even seemingly simple anticoagulant treatment tasks are potentially reorganized and reformulated in an ad-hoc fashion. An example of such a situation is when the sick leave of an anticoagulation nurse causes a disruption in the normative workflow of the guideline assignment process involving distribution of tasks to various personnel. If such a process is supported by an electronic system implemented, for example, as a conventional computer system implementing an anticoagulation task list, the result can be that the system has to be bypassed. This can be caused, for example, by the redistribution of the tasks of the missing anticoagulation nurse among several other members of the personnel instead of a single substituting nurse. Direct person-to-person communication and manual note keeping is the typical fallback position when the normative process is disrupted. This results in additional work as information gathered during the ad-hoc re-formulation has to be augmented by hand back to the electronic system. A common experience is that structured information management tools are discarded in case of a disruption in the workflow. Adaptations to temporary, yet fundamental changes in the anticoagulation workflow are a major hurdle for conventional tools to maintain automation especially as there can be a large number of reformulation combinations.

Considering the anticoagulation nurse model, the reality is that often only a single experienced person (i.e., the professional responsible for it and experienced in it) fully understands, for example, what the appearance of new P-INR measurement to an electronic task list implies. There are a surprisingly large number of decision paths which can be followed depending on the measurement value, on the patient in question, on the time of the day and on other contextual information. Most importantly, much of the knowledge needed in taking the correct actions exists only in tacit form. In order that an anticoagulation nurse can work rationally he or she has to be aware of all the intricacies related to each patient’s status and even the social environment of the primary care clinic. As a concrete example, the simplistic idea that the addition of a time-based watchdog to the electronic follow-up task list to monitor the status of new follow-ups and to notify pre-defined personnel of unattended results fails to capture the complexity of the situation.

Apart from complexities in tasking, the knowledge required for making an informed decision on the next anticoagulation medication dosage is a complexly coupled one. Of course, typically the decision is based on a set of discrete facts (i.e., the measured P-INR value and recent care history). However, as control of blood thinning medication can potentially depend on a very large set of facts the situation is sometimes much more complex. Dental work to be done to the patient, dietary choices and various factors dispersed in numerous sources and even factors related to recent changes in a patient’s personal life have all to be taken into account. The explicit rules required for always arriving at the optimal or even adequate decision can be prohibitively difficult to set down, and especially to maintain, even in the case of a seemingly simple care decision.

## CONCEPTUAL SYSTEM

To illustrate the application of open information management in the described case of anticoagulant treatment follow-up, a conceptual system is presented. Fig. (**1**) shows the architecture of the system.

The engine provides an infrastructure for a communication-oriented anticoagulation tasking and guideline assignment system. The input for the system is new laboratory results and the related decisions of the users. The output of the system includes messages such as treatment guidelines advancing the execution of the decisions made by the users. The main activity of the system is related to the maintenance of task lists of the users. The actions needed for task list manipulation and for a simple user interface with user authentication would be implemented as components running on top of the engine. The initial goal for the system is to get a care decision from a user for each incoming laboratory result.

As discussed in the previous chapter, the explanations connect the goal, the actions and the information to be managed. In the conceptual system, an explanation is a set of concept relations with a given format. The following is an example of a set of concept relations forming an explanation for a laboratory result:

724439 “laboratory result” (for) “patient” 1115393687

724440 *724439c (is) “29021936-1234”

724441 *724439a (is) “P-INR value”

724442 *724441a (is) “2.27”

A concept relation has a syntax similar to the one used in the W3C RDF standards [30]. Specifically, each relation is a <unique identification, A, B, C, timestamp> 5-tuple. Basically, the member B relates the member A and C to each other with an optional timestamp indicating the absolute markup time. The couplings between the concept relations are either implicit or explicit. For example, an implicit coupling exists between relations containing “P-INR value” labeled concepts. Implicit couplings are found through string similarity matching. Explicit couplings are defined through labeling A, B and C members with a reference notation. This notation uses relation sequence numbers and A, B or C membership as points of reference. For example, the member C could be labeled “*724439c”, which indicates a reference to the C member of the relation with identification 724439.

In the initial state, the system has only one possible action it may use on an incoming new laboratory result. That action is the assignment of the laboratory result to the task list of the anticoagulation nurse. Task lists are explanations composed of concept relations. Users access their task list through a user interface mapping a set of explanations into a view presented to the user. The user has the possibility to react on the task list items by firing actions available in the system. The LIS (laboratory information system) interfacer injects new P-INR measurement results into to the set of explanations by transcribing an export provided by the LIS system into a set of concept relations. Similarly, the available actions are described through a set of concept relations with semantic coupling to physical components providing the actual action facilities when actions are fired. An example of such an action is the transfer of a task to the task list of a different user and the sending of an e-mail to that user, for example a physician, indicating the need for him or her to access the task list to submit a treatment guideline to a new P-INR measurement result. Another example of an action is the actual assignment of a guideline.

The related sensor and action interfacer components are implemented as conventionally programmed components wherein the semantic couplings between physical actions and sensors are coupled to their explanations with the help of metadata facilities provided by the programming environment. Only the generic parts of the system would have to be conventionally programmed. All parts of the system specific to anticoagulant treatment follow-up reside in explanations composed of concept relations. This division is one of the key issues enabling system evolution during use and, to some extent, without computer programming capabilities.

Considering the LT and ST labels in the explanations component of Fig. (**1**), they indicate a distinction between long-term memory and short-term memory in the set of concept relations constituting the explanations. Explanations nearer to long-term memory represent a collection of concepts and their explicit couplings describing relatively static, commonly accepted notions of fact. Primarily long-term memory explanations include those related to a generic common sense as well as those for health care and primary care in general and for anticoagulant treatment follow-up in particular. The short-term memory primarily contains explanations related to the observed operation of the concept demonstration system. The explanations describing the task list are nearer the ST end of the spectrum. The explanation organizer distills explanations from the short-term memory to the long-term memory. The distillation is based on an analysis of repeatedness and other factors in the short-term explanations generated by the management of anticoagulation tasks and results in higher-abstraction level concept relations. The distillation is supervised by a user. As an example of the evolution of the system, the processing of the incoming new laboratory results could be broadened from the initial state to include the selection of the user to whose task list the new result is assigned. The motivation for the selection would be to assign the laboratory results indicating abnormal values directly to the task list of the physician currently assigned for the patient in question. The introduction of this new functionality would not require any modifications to programmed components. Only the explanations were augmented.

Considering the potential capabilities of the presented approach, the fact that functionality-providing facilities of the system are accessed through natural language-coupled interfaces gives the possibility to augment the physical capabilities (i.e., to add sensors and actions) of the system in an accessible way. For example, a new action for sending mobile short messages would be implemented by adding explanations for the action interface to the set of explanations and deploying the corresponding physical component. The loose nature of the related couplings would enable doing this in real time without disturbing the use of the system. Concerning the decision support capabilities of the system, the system can provide for the recording of complex decisions made by professionals since all user activity is stored as explanations. The resulting knowledge-base of explanations provides a set of explained paths for the personnel to take in the future.

## CONCLUSIONS

It can be argued that the introduced concept of open information management is in many ways orthogonal to certain preconceptions related to the design, implementation and use of information technology. The motivation for our approach comes from the recent scientific discussion on the nature of contemporary clinical work and from the related reported shortcomings of information technology. Considering the promises which can be deduced from the characteristics of open information management, it must be noted that this paper presents only a conceptual framework for future engineering research. There are no guarantees that a practical implementation will not lead into insurmountable technical challenges. For example, constructing an initial set of actions with the accompanying explanations up to a useful level may prove to be laborious. Related to this, the user effort required to accomplish information management automation with at least some utility has to be reasonable.

Considering the role of users in the combined design-and-use process of open information management, it may seem that we are proposing to move the engineering task to the busy clinician. However, our hypothesis is that the technology allows both the engineer and the user concentrate on what they are best at. For example, one common point of friction is the boundary between the application developer and the user. It is often difficult to transfer the factual needs of users, and the optimal solutions to these needs, to application functionality due to a lacking common ground. Our hypothesis is that ultimately the presented approach enables the engineer to concentrate on infrastructural tasks while the user, as a by-product of primary work, provides the necessary feedback to contribute to the evolution of tools, which is collectively cumulative. One example of this would be incrementally and integrally built electronic decision support functionality.

Considering our proposal to use natural language words for denoting concepts within explanations, we must emphasize that we are not targeting a system that can autonomously interpret textual compositions. However, we estimate that free text compositions provided by users can probably be utilized to accelerate the complementation of explanations. For example, simple key word searching may provide a tool to prioritize among choices prompted to the user.

An important aspect of information management which we have not addressed is security. We estimate that current technological approaches for managing security can be continued to be leveraged. However, an argument can be made that the open information management paradigm enables an approach to security issues where, for example, access constraints are expressed with the same types of explanations as are other aspects of operation. This means that security does not have to be something seen as an infrastructural entity requiring more or less separate administration.

The next concrete step in this line of research is the practical implementation of a system incorporating the presented paradigm in a specific application area, namely the one presented in the previous section.

## Figures and Tables

**Fig. (1).**
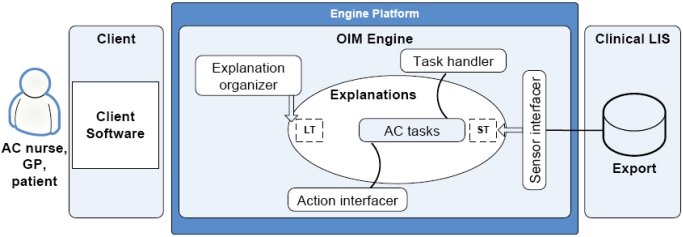
Conceptual system architecture.
